# Use of analgesics/antipyretics in the management of symptoms associated with COVID-19 vaccination

**DOI:** 10.1038/s41541-022-00453-5

**Published:** 2022-03-02

**Authors:** Eng Eong Ooi, Arti Dhar, Richard Petruschke, Camille Locht, Philippe Buchy, Jenny Guek Hong Low

**Affiliations:** 1 Program in Emerging Infectious Diseases, Duke-National University of Singapore Medical School, Singapore, Singapore; 2 Yong Loo Lin School of Medicine, National University of Singapore, Singapore, Singapore; 3 Saw Swee Hock School of Public Health, National University of Singapore, Singapore, Singapore; 4 Medical Affairs, GlaxoSmithKline, Singapore, Singapore; 5 Medical Affairs, GlaxoSmithKline, Warren, NJ USA; 6 Univ. Lille, CNRS, Inserm, CHU Lille, Institut Pasteur de Lille, U1019 - UMR 8204 - CIIL - Center for Infection and Immunity of Lille, Lille, France; 7 Department of Infectious Diseases, Singapore General Hospital, Singapore, Singapore

**Keywords:** Diseases, Immunology

## Abstract

COVID-19 vaccines are effective and important to control the ongoing pandemic, but vaccine reactogenicity may contribute to poor uptake. Analgesics or antipyretic medications are often used to alleviate vaccine side effects, but their effect on immunogenicity remains uncertain. Few studies have assessed the effect of analgesics/antipyretics on vaccine immunogenicity and reactogenicity. Some studies revealed changes in certain immune response parameters post-vaccination when analgesics/antipyretics were used either prophylactically or therapeutically. Still, there is no evidence that these changes impact vaccine efficacy. Specific data on the impact of analgesic/antipyretic medications on immunogenicity of COVID-19 vaccines are limited. However, available data from clinical trials of licensed vaccines, along with recommendations from public health bodies around the world, should provide reassurance to both healthcare professionals and vaccine recipients that short-term use of analgesics/antipyretics at non-prescription doses is unlikely to affect vaccine-induced immunity.

## Introduction

The coronavirus disease 2019 (COVID-19) pandemic, caused by the emergence of severe acute respiratory syndrome coronavirus 2 (SARS-CoV-2), has impacted global health and economies. Millions have died from this illness and many more have experienced reduced quality of life from COVID-19 ailment and the disease measures enforced to control virus transmission. Thus, the arrival of efficacious vaccines has been much welcomed.

To date, over 300 vaccine candidates have emerged, with more than 100 currently in clinical development and 17 deployed for emergency use in various countries around the world^1,2^. Among them, eight vaccines have currently been granted emergency use authorization by the World Health Organization (WHO), including messenger ribonucleic acid (mRNA), adenovirus vector-based, and inactivated virus vaccines^2^. Many other vaccines are at various stages of clinical development and consideration for emergency use authorization.

Within 6 months of the first COVID-19 vaccine approval^3^, over 2 billion doses of vaccines have been administered worldwide^4^. Despite this unprecedented pace in vaccine rollout, many more individuals need to be vaccinated and boosted before the necessary beneficial epidemiological impact of vaccination can be achieved and sustained globally. As vaccine supply increases, greater willingness to be vaccinated will also be required to increase uptake. One reason for vaccine hesitancy is the concern for vaccine-associated side effects^5^, including pain at the injection site, headache, myalgia, fever, and fatigue, although they are usually transient. Thus, therapeutic use of medication to reduce the rate and/or severity of vaccine-induced side effects, without impacting vaccine immunogenicity and efficacy, may be helpful in countering such hesitancy and improving vaccine and booster uptake rates.

Analgesics and antipyretics, such as acetaminophen (paracetamol) and non-steroidal anti-inflammatory drugs (NSAIDs, such as ibuprofen), have been routinely used for decades to manage acute side effects, or reactogenicity, following vaccination. However, some have speculated that the use of antipyretics/analgesics to manage reactogenicity may impact vaccine efficacy. As booster vaccinations will eventually be needed to sustain vaccine effectiveness, there is an urgent need to determine whether the use of over-the-counter analgesics/antipyretics has any negative impact on vaccine immunogenicity and efficacy. Furthermore, it is important to determine whether vaccine immunogenicity and reactogenicity following the use of these medications vary by age group.

This article summarizes the evidence on the use of analgesic/antipyretic medications to relieve side effects following vaccination. It describes both the clinical and molecular bases of how such medications can minimize side effects without compromising vaccine immunogenicity and efficacy. Finally, it discusses the implications of such interventions for the global vaccination efforts against COVID-19.

## Background on immunogenicity and reactogenicity of vaccines

Immune responses and the effectiveness of vaccines differ throughout life. At birth, the child’s immune system is considered ‘immature’, and maternal antibodies transferred trans-placentally and by breast feeding can interfere with vaccination. Consequently, many childhood vaccines are administered after several months of age, when the immune system has matured and maternal antibodies have waned. However, even in the absence of maternal antibodies, increasing age has been associated with better vaccine efficacy. Indeed, dengue vaccination in younger children between the ages of 2 and 6 years old showed lower vaccine efficacy than older children, even though both groups were immunologically naïve to dengue at baseline^6,7^. The effectiveness of measles vaccination between 12 and 18 months of age is another good example of this phenomenon^8^. With aging, the thymus involutes and naïve T cell production declines which, together with other age-related changes, leads to immunosenescence^9^. Comorbidities can also impact immune responses and affect vaccine efficacy in the elderly^10^. Thus, immunological findings from different vaccine studies may not be broadly applicable across all age groups.

Vaccines contain viral antigens or viral genes for translation into antigens, preferably in antigen-presenting cells upon inoculation, to induce specific immune responses and memory to protect against the corresponding pathogen and associated disease^11^. Vaccination first induces the innate immune response, which includes phagocytosis, release of chemokines and cytokines, complement activation, and cellular recruitment^11^. These innate immune responses play fundamental roles in programming subsequent adaptive immune responses^12^ and are thus required for the development of acquired immunity^13^. Among the cells in the innate immune system are antigen-presenting cells, such as dendritic cells^12,14^, which are essential in linking between the innate and adaptive immune systems. These cells interact with T and B cells to trigger the long-lasting adaptive responses^12,15^.

As the innate immune response is key to induce adaptive immunity, it has been widely assumed that vaccine-associated side effects are correlated with immunogenicity. However, findings from recent studies with the live attenuated yellow fever vaccine suggest that this assumption is not applicable for all vaccines. Studies on yellow fever vaccine have shown that individuals who developed side effects had higher levels of endoplasmic reticulum (ER) stress response genes expressed in the blood at baseline, likely in lymphocytes, as well as lower level of tricarboxylic acid cycle activity, likely in the monocytes^16^. As vaccination induces protein expression, the ER stress would be worsened and further complicated by increased energy demand, resulting in maladaptive ER stress response and pro-inflammatory response. This early pro-inflammatory response is directly correlated with the development of side effects, such as headache, myalgia, and fever^11,16^. In contrast, the innate immune response that correlates with adaptive immunogenicity develops from 3 to 7 days post-vaccination, with type-I interferon responses featuring more prominently than pro-inflammation^17,18^. Indeed, Chan et al.^16^ found no difference in neutralizing antibody levels against the yellow fever virus between participants who developed side effects and those who did not. Thus, there could be qualitative and temporal differences in the innate immune response that are involved in the development of side effects versus those that shape adaptive immunity. However, the yellow fever vaccine is a live attenuated virus; the separation between the innate immune responses that drive side effect manifestation and adaptive immune responses may be less distinct in other forms of vaccines. Hence, whether side effects can be minimized by analgesics and antipyretics without compromising adaptive immune responses will still need to be gleaned from clinical observations.

## Effect of analgesic and antipyretic medicines on immune responses to vaccination

Although analgesics and antipyretics are not indicated for prophylactic use, they can be administered at the time of vaccination to prevent side effects or therapeutically following side-effect onset. A limited number of studies have evaluated the effect of analgesics/antipyretics on immunogenicity^11,19–22^. However, these studies have been limited to either specific age groups of the population or specific vaccines. As COVID-19 vaccination is being applied universally and eventually to all age groups, we undertook a more expansive review of the literature on acetaminophen and NSAIDs and their effects on vaccination.

### Acetaminophen (Paracetamol)

Despite its widespread use for many years, the mechanism(s) of action of acetaminophen remain(s) unclear. The drug may exert its therapeutic effects through inhibition of cyclo-oxygenase (COX)-1 and −2 and the subsequent decrease of prostaglandin synthesis^23^, although other mechanisms have been suggested. Both COX-2 activation and prostaglandin production are important in innate immune response signaling^23^. Activated T and B cells express COX-2 and produce prostaglandins^24^. A small number of randomized controlled trials with several different vaccines have investigated the effects of prophylactic or therapeutic acetaminophen on vaccine responses in pediatric^25–28^ and adult populations^29^, with conflicting results (Table 1).

**Table 1:** Randomized clinical trials of analgesic and antipyretic medicines: effect on immune responses to vaccination.

Trial/design	Vaccine	Analgesic/antipyretic	Immunogenicity	AE
**Childhood**				
Prymula et al.^26^ *N* = 459 Age: 16 weeks at enrollment, 12–15 months at booster	PHiD-CV, DTaP-HBV-IPV/Hib, and oral human rotavirus (live attenuated)	**Acetaminophen** (3 doses based on body weight*) every 6–8 h for 24 h versus no acetaminophen (control)	Significant reduction in antibody GMC with prophylactic acetaminophen versus no acetaminophen for pneumococcus vaccine serotypes, Hib, diphtheria, tetanus, and pertussis (reduced by 27–55% vs no prophylaxis) Post-booster, lower antibody GMC persisted for most pneumococcal vaccine serotypes and tetanus with prophylactic acetaminophen at both primary and booster vaccinations versus no acetaminophen (reduced by 21–45% vs no prophylaxis)	Control versus acetaminophen: fever (66% vs 42%) following primary and (58% vs 36%) booster vaccination
Prymula et al.^27^ *N* = 220 (previously primed and boosted in Prymula et al.^26^ + *N* = 223 unprimed age-matched controls	PHiD-CV	**Acetaminophen** (3 doses based on body weight*) every 6–8 h for 24 h versus no acetaminophen (control)	Antibody GMC lower with prophylactic acetaminophen versus no acetaminophen for serotypes 1 (–41%), 4 (–31%), 7 F (–35%), and 9 V (–37%)	Not reported
Age: 4 years				
Sil et al.^28^ *N* = 975 Age: 6–8 weeks	DTwP-HBV-Hib	Post-hoc analysis according to **acetaminophen** use: prophylactic or treatment (dose and frequency not stated) or no acetaminophen (control)	No difference in antibody GMC according to acetaminophen use (therapeutic or prophylactic) versus control	Not reported
Falup-Pecurariu et al.^25^ *N* = 812 Age: 12–16 weeks	PHiD-CV, DTaP-combined (DTaP-HBV-IPV/Hib and DTaP-IPV/Hib)	**Ibuprofen** (10 mg/kg, max dose 30 mg/kg) or **acetaminophen** (15 mg/kg, max dose 60 mg/kg), three doses every 6–8 h started immediately (at 0 h) or delayed (at 4-6 h) versus no ibuprofen or no acetaminophen (controls)	No clinically relevant impact of immediate or delayed prophylactic administration of ibuprofen on antibody GMC during primary or booster vaccination Prophylactic acetaminophen at primary vaccination reduced post-primary and post-booster antibody GMC, but had no detrimental effect on immunogenicity when administered at booster dose only	Control versus ibuprofen: no statistically significant reduction in fever (61.4% vs 49.7–62.6%) Control versus acetaminophen: decreased fever incidence (54.1% vs 32.9–38.0%)
Walter et al.^31^ *N* = 142 Age: 6–47 months	Inactivated influenza	**Ibuprofen** (~10 mg/kg) every 6–8 h for 24 h or **acetaminophen** (15 mg/kg) every 4–8 h for 24 h or placebo (control)	No statistically significant differences in GMC between either of the treatment groups and placebo	Control versus acetaminophen and ibuprofen: no significant differences on Day 0 (0%, 4.3%, 0%) or Day 1 (0%, 1.9%, 0%)
Wysocki et al.^30^ *N* = 908 Age: 2 months	PCV13, combined DTaP-HBV-IPV/Hib	**Acetaminophen** (15 mg/kg) or **ibuprofen** (10 mg/kg) every 6–8 h either immediately (at 0, 6–8, and 12–16 h) or delayed (at 6–8 and 12–16 h) versus no acetaminophen or ibuprofen (control)	Antibody GMC for 5/13 pneumococcal serotypes significantly lower with acetaminophen prophylaxis versus controls at 5 months No effect of ibuprofen on pneumococcal responses, but significant reduction in antibody response to pertussis and tetanus antigens at 5 months when started at vaccination	Fever on Day 0 was lowest with antipyretic prophylaxis; in participants receiving delayed antipyretics, fever rates were similar to controls. Acetaminophen recipients reported fever less frequently than ibuprofen recipients
**Adult**				
Aoki et al.^29^ *N* = 262 Age: >18 years	Inactivated trivalent whole-virus influenza	**Acetaminophen** (325 or 650 mg) every 4 h at 0, 4, 8, and 12 h versus placebo (control)	Antibody GMC similar for both acetaminophen dosing groups and placebo at all times	Acetaminophen versus control: incidence of sore arm: 25–28% lower; incidence of nausea: 90% lower
Lafferty et al.^32^ *N* = 40 Age: elderly	Pneumococcal	**Indomethacin** (25 mg/day for 5 days post-vaccination versus no indomethacin (control)	No significant difference in mean increases in antibody GMC to 12 pneumococcal polysaccharide types for indomethacin versus control groups	Not evaluated
Agarwal et al.^33^ *N* = 67 (*n* = 131 vaccinations)	Inactivated trivalent whole-virus influenza	Long-term **NSAID** use (no further details on dosing provided) versus non-users (control)	No significant increases in antibody titer between NSAID users and non-users post-vaccination	Not evaluated
Age: ≥65 years				
Jackson et al.^34^ *N* = 1,597	Monovalent H1N1 influenza	Low-dose **aspirin** (no further details on dosing provided) versus non-users (control)	Antibody GMC was higher in users of low-dose aspirin than in non-users (133.8 vs 119.2) at Day 21, but this difference was not statistically significant and remained insignificant after adjustment for factors including vaccine, age, concomitant medication, and comorbidities	Not evaluated
Age: ≥50 years				

*80 mg per administration (53.3–34.3 mg/kg/24 h) for infants weighing between 4.5 kg and less than 7 kg, and 125 mg per administration (≤53.6 mg/kg/24 h) for infants weighing 7 kg or more. At booster vaccination, the same dose was given to infants weighing between 7 kg and less than 9 kg and those with bodyweight of 9 kg or greater received four administrations of 125 mg acetaminophen each within 24 h (≤55.6 mg/kg/24 h).

*AE* adverse event, *DTaP-HBV-IPV/Hib* hexavalent diphtheria-tetanus-3-component acellular pertussis-hepatitis B-inactivated poliovirus types 1, 2, and 3 *Haemophilus influenzae* type b, *DTaP-IPV/Hib* diphtheria-tetanus-acellular pertussis-inactivated poliomyelitis-adsorbed conjugated *Haemophilus influenzae* type b, *DTwP-HBV-Hib* diphtheria-tetanus-whole cell pertussis-hepatitis B recombinant-inactivated poliomyelitis-adsorbed conjugated *Haemophilus influenzae* type b, *GMC* geometric mean concentration, *Hib Haemophilus influenzae* type b, *NSAID* non-steroidal anti-inflammatory drug, *PCV13* 13-valent pneumococcal conjugate vaccine, *PHiD-CV* ten-valent pneumococcal non-typeable *Haemophilus influenzae* protein D-conjugate vaccine.

In a non-inferiority study investigating the use of prophylactic acetaminophen on vaccine immunogenicity, Falup-Pecurariu et al.^25^ observed a statistically non-significant trend for reduced antibody titers when prophylactic acetaminophen was given to infants immediately or several hours after the first dose of pneumococcal non-typable *Haemophilus influenzae* protein D conjugate vaccine (PHiD-CV) co-administered with diphtheria-tetanus-acellular pertussis-hepatitis B recombinant-inactivated poliomyelitis-adsorbed conjugated *Haemophilus influenzae* type b vaccine (DTaP-HBV-IPV/Hib) and diphtheria-tetanus-acellular pertussis-inactivated poliomyelitis-adsorbed conjugated *Haemophilus influenzae* type b vaccine (DTaP-IPV/Hib), compared with infants who did not receive prophylactic acetaminophen. This trend was not, however, seen after a booster dose^25^. Similarly, in children receiving PHiD-CV co-administered with DTaP-HBV-IPV/Hib and oral human rotavirus vaccines, prophylactic acetaminophen given immediately after vaccination (three doses at 6–8 h intervals) was associated with lower antibody titers but not lower seroconversion rates compared with those not receiving acetaminophen. The impact was less marked following booster vaccination^26^. However, this effect did not impact subsequent induction of immunological memory^27^.

Data from infants given the diphtheria-tetanus-whole cell pertussis-hepatitis B recombinant-inactivated poliomyelitis-adsorbed conjugated *Haemophilus influenzae* type b (DTwP-HBV-Hib) combination vaccine found that immune responses were similar regardless of whether acetaminophen was used prophylactically, therapeutically, or not at all^28^.

The impact of acetaminophen prophylaxis on vaccine immunogenicity may be antigen-specific^30^. Among infants who received repeat doses of the 13-valent pneumococcal conjugate (PCV13) and combined DTaP-HBV-IPV/Hib vaccines, antibody titers for five of 13 pneumococcal serotypes were significantly lower at 5 months of age among participants given prophylactic acetaminophen than among those who did not receive acetaminophen^30^. No difference in antibody titer was observed after booster doses at 13 months of age.

There may also be age-specific effects on acetaminophen prophylaxis and immunogenicity. A randomized, double-blind, placebo-controlled study in 474 Canadian healthcare workers found that acetaminophen given immediately after influenza vaccine administration (four doses at 4-h intervals) did not affect antibody responses^29^.

In summary, research on the effect of acetaminophen on vaccine immunogenicity is limited and does not establish clear evidence against the use of acetaminophen as a treatment for vaccine-induced side effects. Although the clinical significance of prophylactic acetaminophen use on antibody level is not known, some data suggest that the use of therapeutic acetaminophen may be preferred over prophylactic use to avoid any potential impact on immunogenicity.

### NSAIDS

NSAIDs, most commonly ibuprofen at non-prescription doses, are also often used to manage post-vaccination side effects. The therapeutic effects of NSAIDs are the result of the inhibition of well-defined inflammatory pathways involving prostaglandin synthesis and COX-1 and COX-2 activities. Investigations into the impact of NSAIDs on vaccine immunogenicity have been mostly conducted in children and have led to varying conclusions (Table 1).

Neither immediate nor delayed prophylactic ibuprofen use had an impact on the immune responses to primary or booster vaccination in infants receiving the PHiD-CV vaccine^21^. Similarly, children receiving the inactivated influenza vaccine did not generate lower immune responses if they had received prophylactic ibuprofen, compared with those not receiving ibuprofen^20,31^.

In another study by Wysocki et al.^30^, infants who were given ibuprofen prophylaxis with repeat doses of PCV13 and combined DTaP-HBV-IPV/Hib vaccines had significantly reduced antibody responses to *Bordetella pertussis* filamentous hemagglutinin and the tetanus toxin, but not to pneumococcal antigens^30^.

Early investigations among a small number of older patients indicated that NSAID use is unlikely to impact vaccine responses^32^. More recently, an analysis of patients aged ≥65 years receiving a trivalent inactivated influenza vaccine reported altered antibody production, B cell phenotypic changes, alteration in immune cell proportions, and transcriptome-wide modifications in those receiving long-term NSAID^33^. However, there was no statistically significant difference in antibody titers between NSAIDs users and non-NSAIDs users. A meta-analysis of four clinical studies on long-term aspirin use in older adults (*n* = 1,597) also found no difference in antibody titers following influenza vaccination^34^.

In summary, while some studies have demonstrated a potential impact of ibuprofen prophylaxis on post-vaccination antibody production in children, studies in older adults found no significant impact. The observed changes in antibody titers appear to have little or no clinical impact, since the tested vaccines were effective.

## Reactogenicity of current COVID-19 vaccines

The urgent need for COVID-19 vaccines posed an unprecedented dilemma—the need to expedite preclinical and clinical development of vaccines (some of which were based on relatively novel technologies) without jeopardizing safety and efficacy evaluation. Table 2 summarizes available reactogenicity data for the COVID-19 vaccines currently approved for emergency use.

**Table 2:** Reactogenicity of COVID-19 vaccines approved by WHO for emergency use.

Vaccine	Trial/population	Reactogenicity	
		Injection-site pain	Selected systemic symptoms
**mRNA-based**			
BNT162b2 (Pfizer/BioNTech)^37^	Phase 2/3, OB, PC, R *N* = 18,556 (*n* = 8,183 reactogenicity subset) Age: ≥16 years (median 52 years, range 16–91 years) Males: 51%	Dose 1: 83% (≤55 years); 71% (>55 years) Dose 2: 71% (≤55 years); 66% (>55 years)	Fatigue Dose 1: 47% (≤55 years); 34% (>55 years) Dose 2: 59% (≤55 years); 51% (>55 years) Headache Dose 1: 42% (≤55 years); 25% (>55 years) Dose 2: 52% (≤55 years); 39% (>55 years)
mRNA-1273 (Moderna)_43_	Phase 3, OB, PC, R *N* = 30,420 Age: ≥18 years (mean 51.4 years, range 18–95 years) Males: 52.7%	Dose 1: 83.7% (all); 86.9% (<65 years); 74.0% (≥65 years) Dose 2: 88.2% (all); 89.9% (<65 years); 83.2% (≥65 years)	Fatigue Dose 1: 37.2% (all); 38.4% (<65 years); 33.3% (≥65 years) Dose 2: 65.3% (all); 67.6% (<65 years); 58.3% (≥65 years) Headache Dose 1: 32.7% (all); 35.3% (<65 years); 24.5% (≥65 years) Dose 2: 58.6% (all); 62.8% (<65 years); 46.2% (≥65 years)
**Adenovirus-based**			
AZD1222 (Oxford/AZ)^47^	Phase 2, R, C *N* = 420 Age: ≥18 years (*n* = 100 aged 18–55, *n* = 120 aged 56–69, and *n* = 200 aged ≥70) Males: 50%*	Dose 1: 61% (18–55 years); 43% (56–69 years); 20% (≥70 years) Dose 2: 49% (18–55 years); 34% (56–69 years); 10% (≥70 years)	Fatigue Dose 1: 76% (18–55 years); 50% (56–69 years); 41% (≥70 years) Dose 2: 55% (18–55 years); 41% (56–69 years); 33% (≥70 years) Headache Dose 1: 65% (18–55 years); 50% (56–69 years); 41% (≥70 years) Dose 2: 31% (18–55 years); 34% (56–69 years); 20% (≥70 years)
Ad26.COV2.S (Janssen/J&J)^50^	Phase 3, DB, PC, R *N* = 44,325 Age: ≥18 years (median 52 years, range 18–100 years) Males: 54.9%	48.6% (all); 58.6% (18–59 years); 33.3% (≥60 years)	Fatigue 38.2% (all); 43.8% (18–59 years); 29.7% (≥60 years) Headache 38.9% (all); 44.4% (18–59 years); 30.4% (≥60 years)
**Inactivated virus**			
BBIBP-CorV (Sinopharm)^52^	Phase 2, R, DB, PC *N* = 448 Age: 18–59 years (mean 41.7 years, SD 9.9 years) Males: 45%	16%^‡^	Fatigue: 3%^‡^ Headache: 1%^‡^
CoronaVac (Sinovac Biotech)^53^	Phase 2, R, DB, PC *N* = 600 Age: 18–59 years (mean 42 years) Males: N/A^#^	Doses on Day 0 and 14: Dose 1: 9.2% (3 µg), 16.7% (6 µg) Dose 2: 13.3% (3 µg), 11.8% (6 µg) Doses on Day 0 and 28: Dose 1: 7.5% (3 µg), 10.0% (6 µg) Dose 2: 2.6% (3 µg), 5.9% (6 µg)	Fatigue Doses on Day 0 and 14: Dose 1: 1.7% (3 µg), 5.0% (6 µg) Dose 2: 1.7% (3 µg), 1.7% (6 µg) Doses on Day 0 and 28: Dose 1: 8.3% (3 µg), 1.7% (6 µg) Dose 2: 2.6% (3 µg), 0.9% (6 µg) Headache Doses on Day 0 and 14: Dose 1: 0.8% (3 µg), 0.8% (6 µg) Dose 2: 0.8% (3 µg), 2.5% (6 µg) Doses on Day 0 and 28: Dose 1: 2.5% (3 µg), 0.8% (6 µg) Dose 2: 1.7% (3 µg), 0.0% (6 µg)
CoronaVac (Sinovac Biotech)^54^	Phase 1/2, R, DB, PC *N* = 349 (Phase 2) Age: ≥60 years (mean 66.6 years, SD 4.7 years) Males: 48%	Dose 1: 2% (1.5 µg), 8% (3 µg), 5.1% (6 µg) Dose 2: 9.1% (1.5 µg), 7% (3 µg), 5.1% (6 µg) *Doses were administered on Day 0 and 28*	Fatigue Dose 1: 3% (1.5 µg), 3% (3 µg), 2% (6 µg) Dose 2: 1% (1.5 µg), 0 (3 µg), 1% (6 µg) Headache Dose 1: 0 (1.5 µg), 0 (3 µg), 2% (6 µg) Dose 2: 0 (1.5 µg), 0 (3 µg), 0 (6 µg)

*Based on *N* = 552 in the Phase 2/3 trial. ^‡^AEs after first and second doses combined. ^#^Proportion of male participants was 44% in the Day 0 and 14 vaccination cohort (n = 372) and 49% in the Day 0 and 28 vaccination cohort (*n* = 371) for Phase 1 and 2 combined data. *AE* adverse event, *AZ* AstraZeneca, *C* controlled, *COVID-19* coronavirus disease 2019, *DB* double-blind, *J&J* Johnson & Johnson*, mRNA* messenger ribonucleic acid, *OB* observer-blinded, *PC* placebo-controlled, *R* randomized, *SD* standard deviation, *WHO* World Health Organization.

### mRNA-based vaccines

BNT162b2 (Pfizer-BioNTech) is a lipid nanoparticle-formulated, nucleoside-modified RNA vaccine encoding the full-length spike protein^35,36^. Among 8,183 vaccine recipients included in a reactogenicity subset of a Phase 2/3 study, injection-site pain was the most commonly reported local side effect, affecting 78–83% of recipients after the first dose and 66–71% after the second dose (Table 2)^37^. The most commonly reported systemic symptoms were fatigue (34–59%) and headache (39–52%), which generally occurred 1–2 days after vaccination. Both injection-site pain and systemic events were more commonly reported by younger than older recipients, and systemic events were observed more commonly after the second dose than after the first. Vaccine efficacy did not appear to be significantly affected by age in this Phase 2/3 trial^37^. Hwang et al. assessed the association between local and systemic reactogenicity and humoral immunogenicity following administration of BNT162b2 vaccine to healthy volunteers (*n* = 93)^38^. Association was evaluated using multivariate linear regression with adjustment for age, sex, and use of antipyretics. The study found that grades of local and systemic adverse events were not significantly associated with anti-S1 immunoglobulin G levels, suggesting no direct correlation between these adverse events and humoral immunogenicity.

The possibility that inflammatory response to mRNA vaccination underpins adverse events also raises the possibility that those with inflammatory conditions could have more or accentuated side effects. However, a study by Furer and colleagues did not find any significant difference in the rate or severity of side effects from BNT162b2 vaccination between those with autoimmune inflammatory rheumatic diseases compared to healthy controls^39^. However, it should be noted that most of the subjects with autoimmune conditions in this study were also on immunosuppressive therapy at the time of vaccination. Immunogenicity was impaired in those treated with rituximab, glucocorticoids and other immunosuppressive therapies that act on lymphocytes rather than the more widely used antipyretics/analgesics.

mRNA-1273 (Moderna) is a lipid-nanoparticle encapsulated mRNA vaccine, which expresses the pre-fusion-stabilized spike glycoprotein^40,41^. Similar reactogenicity to BNT162b2 has been reported for mRNA-1273 in both a Phase 1 study^42^ and a Phase 3 trial with 30,420 vaccine recipients^43^. In the Phase 3 trial, injection-site pain occurred in 83.7% of vaccine recipients after the first dose and 88.2% after the second dose (Table 2). Injection-site reactions were mostly mild-to-moderate in severity (grade ≤2) and lasted for a mean of 2.6 and 3.2 days after the first and second doses, respectively. Solicited systemic side effects also occurred more frequently after the second dose (79.4%) than after the first (54.9%), with fatigue and headache most commonly reported after both the first and the second dose and events more severe after the second dose than after the first. Both solicited local and systemic side effects were more common among younger (18 to <65 years of age) than older participants (≥65 years of age). However, studies on the safety of vaccination and immunogenicity in the elderly have largely excluded those who are frail with poorly controlled co-morbidities^44^. Ironically, this population is at high risk of severe COVID-19. These are important gaps in knowledge that need to be addressed urgently.

In summary, the reactogenicity (both local and systemic) associated with mRNA-based vaccines observed in clinical trials is notably higher than that seen with most non-COVID vaccines, which may have a negative effect on second dose compliance.

### Adenovirus vector-based vaccines (OXFORD/ASTRAZENECA [AZ], SERUM INSTITUTE OF INDIA, SKBIO, JANSSEN/Johnson & Johnson [J&J])

AZD1222 (Oxford/AZ) is a replication-defective chimpanzee adenovirus-vectored vaccine expressing the full-length SARS-CoV-2 spike glycoprotein gene^45^. AZD1222 is also registered separately for a vaccine produced by the Serum Institute of India^46^ and by SKBio in South Korea.

In the randomized controlled Phase 2 component of a Phase 2/3 trial of AZD1222 (*n* = 420), the most common solicited local side effects after the first dose of the standard-dose vaccine were injection-site pain (in 61% of patients aged 18–55, 43% aged 56–69, and 20% aged ≥70) and tenderness (in 76%, 67%, and 49% of patients in the respective age groups)^47^. The most common solicited systemic side effects across the respective age groups after the first dose of the standard-dose vaccine were fatigue, headache, feverishness, and myalgia^47^. Local and systemic side effects were reported more commonly by younger than by older recipients and more commonly after the first dose than after the second (Table 2). No severe local side effects were reported, and the severity of systemic side effects was lower after the second than after the first dose. No reactogenicity differences between males and females were reported. The study by Hwang et al. (2021) had also included healthy adults (*n* = 42) who were vaccinated with AZD1222^38^. Results were similar to those for recipients of the BNT162b2 mRNA vaccine, with no indication of an association between either local or systemic reactogenicity and humoral immunogenicity.

Ad26.COV2.S (Janssen/J&J) is a recombinant, replication-incompetent human adenovirus type 26 (Ad26) vector encoding a full-length, membrane-bound SARS-CoV-2 spike protein in a prefusion-stabilized conformation^48,49^. Ad26.COV2.S is administered as a single dose, differentiating it from the other vaccines considered here. In a Phase 3 trial (*n* = 44,325), the reactogenicity of the vaccine was reported to be generally mild-to-moderate and transient^50^. The most common local side effect was injection-site pain (48.6%). The most common systemic side effects were headache (38.9%) and fatigue (38.2%) (Table 2). Incidence rates for both local and systemic side effects were higher among individuals aged 18–59 years than among those aged ≥60, and antipyretic use was more frequent in younger than in older vaccine recipients.

### Inactivated Virus Vaccines (SINOPHARM, SINOVAC BIOTECH)

BBIBP-CorV (Sinopharm) is a beta-propiolactone-inactivated SARS-CoV-2 vaccine developed in China and is produced in African green monkey kidney cells (Vero cells) that have been inoculated with SARS-CoV-2 (HB02 strain)^51^. In a Phase 2 study with 448 adults, the most common local side effect after first and second vaccinations was injection-site pain (16%)^52^. The most common systemic side effects were fatigue (3%) and fever (2%). Reactogenicity of the vaccine was generally mild-to-moderate. However, data in the elderly population are limited, as the study only enrolled patients aged 18–59 years.

CoronaVac (Sinovac Biotech) is a formalin-inactivated SARS-CoV-2 vaccine available for emergency use and was also developed in China. It is produced in Vero cells that have been inoculated with SARS-CoV-2 (CN02 strain). Data from Phase 1/2 studies in adults aged 18–59 years^53^ and ≥60 years^54^ indicated mild reactogenicity after vaccination. In younger vaccine recipients (Phase 2 data)^53^, injection-site pain was the most commonly reported side effect, occurring in 21–26% of recipients after both doses when administered 2 weeks apart and in 10–11% when the dosing interval was increased to 4 weeks. Fatigue and headache were the most common systemic side effects (Table 2). The incidence of all side effects was lowest after the second dose. Most were mild and resolved within 48 h. An indirect comparison with data from older recipients^54^ indicated a lower incidence of local and systemic side effects in the older population (Table 2).

### Third (BOOSTER) dose of COVID-19 vaccines

Several countries have now begun to administer a third or booster dose several months after completion of the primary two-dose vaccination. Early data from studies that have explored the benefit of booster vaccination suggest that the safety profile of the booster dose is comparable across the different vaccines, regardless of what vaccine was used for the primary vaccination series^55^. This study, however, did not include the inactivated vaccines.

## Effect of analgesic and antipyretic medicines on immune responses to COVID-19 vaccination

Only one study has specifically addressed the effect of the analgesic and antipyretic medication on the immunogenicity and reactogenicity of COVID-19 vaccines^56^. However, in most of the other COVID-19 vaccine trials, participants were allowed to use analgesics and antipyretics to treat post-vaccination symptoms. Where available, data from these studies offer some insight into the potential impact of these medicines on immune responses to COVID-19 vaccines (Table 3).

**Table 3:** Use of analgesic/antipyretic medication on symptoms following COVID-19 vaccination in clinical trials.

Vaccine	Trial	Analgesics/antipyretics		
		Treatment/prophylaxis	Use (% patients)	Effect on vaccine reactogenicity
BNT162b2 (Pfizer/BioNTech)	Phase 1 *N* = 90^57^	Treatment: antipyretic/pain medication following vaccination (not specified)	Use of antipyretic/pain medication increased with increasing dose level and number of doses administered in both age groups (data not shown)	—
	Phase 2/3 *N* = 8,183 (reactogenicity subset)^37^		Aged 16–55 years Dose 1: 28% versus 14% for placebo Dose 2: 45% versus 13% for placebo Aged >55 years Dose 1: 20% versus 12% for placebo Dose 2: 38% versus 10% for placebo	—
AZD1222 (Oxford/AZ)	Phase 1/2 *N* = 543^56^	Prophylaxis: acetaminophen (1 g) before vaccination and continued every 6 h for 24 h	10.3%*	Acetaminophen versus no acetaminophen prophylaxis Local AEs • Injection-site pain (50% vs 67%) • Tenderness (77% vs 83%) Systemic AEs • Fatigue (71% vs 70%) • Headache (61% vs 68%) • Muscle ache (48% vs 60%) • Malaise (48% vs 62%) • Chills (27% vs 56%) • Feeling feverish (36% vs 51%)
Ad26.COV2.S (Janssen/J&J)	Phase 3^50,58^	Treatment: antipyretic/pain medication following vaccination (not specified)	All: 19.9% versus 5.7% for placebo Aged 18–59 years: 26.4% versus 6.0% for placebo Aged ≥60 years: 9.8% versus 5.1% for placebo	—

*A protocol amendment permitted prophylactic acetaminophen prior to vaccination at two of five trial sites with participants advised to continue with a 1-g dose of acetaminophen every 6 h for 24 h to reduce vaccine-associated reactions. *AE* adverse event, *AZ* AstraZeneca, *COVID-19* coronavirus disease 2019, *J&J* Johnson & Johnson.

Phase 2/3 data on BNT162b2 (Pfizer-BioNTech) showed that younger vaccine recipients were more likely to use antipyretic or pain medication than older recipients and both age groups were more likely to use these medications following vaccination with BNT162b2 than placebo (Table 3)^37^. Vaccine efficacy against confirmed COVID-19 was 95.0% for onset at ≥7 days after the second vaccination with similar efficacy (generally 90–100%) observed across subgroups defined by age, sex, race, ethnicity, baseline body mass index, and the presence of coexisting conditions^37^. In the Phase 1 part of the trial, analgesic/antipyretic use was reported more frequently with increasing dose and number of doses^57^.

In two of five trial sites participating in a Phase 1/2 study of AZD1222 (Oxford/AZ), a protocol amendment permitted prophylactic acetaminophen prior to vaccination^56^. Vaccine reactogenicity (both local and systemic side effects) was generally reduced in recipients of prophylactic acetaminophen compared with those who did not receive acetaminophen (Table 3). Adjusted analysis of the effect of prophylactic acetaminophen in the first 2 days after vaccination on side effects of any severity demonstrated significant reductions in pain (odds ratio [OR] 0.41), feeling feverish (OR 0.47), chills (OR 0.28), muscle ache (OR 0.51), headache (OR 0.47), and malaise (OR 0.53; all *p* < 0.05). Immunogenicity was not affected.

Data from the Phase 3 study of Ad26.COV2.S (Janssen/J&J) also showed that younger vaccine recipients were more likely to use antipyretic or pain medication than older recipients (Table 3)^50,58^. Vaccine efficacy against severe–critical COVID-19 was 76.7% for onset at ≥14 days and 85.4% for onset at ≥28 days post-vaccination and was considered similar in participants aged ≥60 years and the overall study population, irrespective of sex, race, or ethnicity.

To date, there are no published data on the use of analgesic/antipyretic medication with mRNA-1273 (Moderna), BBIBP-CorV (Sinopharm), or Coronavac (Sinovac Biotech) vaccines.

## Clinical implications/summary

Analgesic and antipyretic medications have been used for decades to manage side effects caused by different types of vaccines in pediatric and adult populations, including the elderly. It is important to distinguish between changes in immune responses and clinical impact, such as vaccine efficacy. While some changes in immune responses (primarily with polysaccharide vaccines) may result from the use of these medications, there is no evidence that their use to manage side effects has any clinically meaningful impact on vaccine efficacy. In fact, it is unlikely that, based on the evidence available in the literature, the short-term use of analgesics/antipyretics at over-the-counter doses suppresses the clinical impact of vaccine-induced immune responses. However, it should also be noted that all of the studies examining the effect of analgesics/antipyretics on immunogenicity have focused exclusively on humoral responses. To our knowledge, none has systematically examined the impact of analgesics/antipyretics on cellular immune responses. Eliciting cellular immunity from COVID-19 vaccination is of particular importance since T cells play a major role in protection against severe and even symptomatic infection^59,60^.

An aspect that remains unknown is how effective analgesics/antipyretics would be in preventing rare but serious adverse events such as myocarditis/pericarditis and thrombosis^61–64^. As inflammation, at least in part, contributes to these serious adverse events^65,66^, anti-inflammatory treatments should also reduce the risk of such rare events. A global collaborative effort will be needed if we are to test prophylactic approaches to prevent such rare but serious events.

Available data from studies of vaccines currently approved for emergency use have provided some insight; efficacy rates of the mRNA vaccines remain very high, despite up to one-fifth of vaccine recipients reporting use of analgesic or antipyretic medication. Public health authorities also continue to recommend the use of these medicines to treat post-COVID-19 vaccination symptoms (Table 4). Any restriction of the use of analgesic and antipyretic medicines in this setting could contribute to COVID-19 vaccine hesitancy, which would have substantial global public health implications.

**Table 4:** Recommendations on analgesic/antipyretic medication for the relief of symptoms following COVID-19 vaccination from various health authorities and organizations.

Health authority/ organization	Recommendation	Last updated	Source
UK National Health Service	Take painkillers such as acetaminophen if needed	March 5, 2021	https://www.nhs.uk/conditions/coronavirus-covid-19/coronavirus-vaccination/safety-and-side-effects/
US Centers for Disease Control and Prevention	Over-the-counter medicines, such as ibuprofen, acetaminophen, aspirin, or antihistamines can be taken to relieve post-vaccination side effects if there are no other medical reasons that prevent taking these medications normally	May 25, 2021	https://www.cdc.gov/coronavirus/2019-ncov/vaccines/expect/after.html
Australia Department of Health	If you experience pain at the injection site or fever, headaches, or body aches after vaccination, you can take acetaminophen or ibuprofen	March 5, 2021	https://www.health.gov.au/sites/default/files/documents/2021/03/covid-19-vaccination-after-your-pfizer-comirnaty-vaccine-covid-19-vaccination-after-your-pfizer-comirnaty-vaccine.pdf
Singapore Ministry of Health	Acetaminophen 1 to 2 tablets every 6 h as needed	February 18, 2021	https://www.moh.gov.sg/covid-19/vaccination
World Health Organization	Take acetaminophen or other painkillers if you develop side effects such as pain, fever, headache, or muscle aches after vaccination	June 22, 2021	https://www.who.int/emergencies/diseases/novel-coronavirus-2019/covid-19-vaccines/advice

*COVID-19* coronavirus disease 2019.
